# Whole-Genome Sequencing and Antimicrobial Resistance Analysis of Enterotoxigenic *Escherichia coli* F5 and F5-F41 Strains Isolated from Neonatal Calves in Inner Mongolia, China

**DOI:** 10.3390/ani16010151

**Published:** 2026-01-05

**Authors:** Mengyuan Xie, Hewei Shang, Liangliang Lv, Pingping Liu, Wenhao Li, Dong Wang, Yue Yuan, Tianqu Huang, Xiumin Wang, Xiaojing Xu

**Affiliations:** 1 College of Veterinary Medicine, Inner Mongolia Agricultural University, Hohhot 010010, China; 18330018791@emails.imau.edu.cn (M.X.); shw@emails.imau.edu.cn (H.S.);; 2 Key Laboratory of Clinical Diagnosis and Treatment Technology in Animal Disease, Ministry of Agriculture and Rural Affairs, Hohhot 010010, China; 3 Hinggan League Animal Disease Prevention and Control Center, Hinggan League 137400, China; 4 Beijing Center Biology Co., Ltd., Beijing 102600, China

**Keywords:** Enterotoxigenic *Escherichia coli*, whole-genome sequencing, antibiotic resistance genes

## Abstract

Enterotoxigenic *Escherichia coli* (ETEC)-induced neonatal calf diarrhea (NCD) causes significant economic losses to the cattle industry; therefore, understanding its antibiotic resistance is crucial for developing targeted prevention and treatment strategies. However, reports on antibiotic resistance in bovine ETEC are currently limited. This study conducted whole-genome sequencing (WGS) and antimicrobial susceptibility testing on ETEC F5- and F5-F41-positive strains isolated from neonatal calf diarrhea samples in Inner Mongolia, China. The results showed that both ETEC F5- and ETEC F5-F41-positive strains are multidrug-resistant, containing ceftriaxone (CRO), ceftazidime (CAZ)) and ciprofloxacin (CIP), which are listed as the highest priority critically important antimicrobial (HP-CIAs) by the World Health Organization (WHO). Combined analysis using the Comprehensive Antibiotic Resistance Database (CARD) and ResFinder 4.1 predictive analysis revealed that the chromosomes and plasmids of the 2 ETEC-positive strains contained 11 classes of antibiotic resistance genes, with the top 3 categories in terms of the number of resistance genes being aminoglycosides, β-lactamases, and chloramphenicols. In addition, various bacterial efflux pumps, including RND, MFS, SMR, and the ABC efflux pump family, were detected. A total of 74 antimicrobial resistance genes were identified in the 2 strains, belonging to 5 categories of drug resistance mechanisms; the antimicrobial resistance phenotype was consistent with the genotype. This study provides a reference for the prevention and treatment of diarrhea caused by ETEC.

## Simple Summary

Enterotoxigenic *Escherichia coli* not only seriously harms the economic and healthy development of the cattle industry but also serves as a reservoir of various antibiotic resistance genes, posing a threat to human public health. This study analyzed the antibiotic resistance spectrum and mechanisms of two pathogenic bacteria. The results showed that both strains were multidrug-resistant, resistant to third-generation cephalosporins, fluoroquinolones, and aminoglycosides. Furthermore, both strains contained 11 classes of antibiotic resistance genes and 74 resistance genes related to various bacterial efflux pumps, including 4 families, across 5 resistance mechanisms in their chromosomes and plasmids. The emergence of multidrug-resistant strains limits clinical antibiotic treatment options, and the widespread dissemination of their resistance genes poses a potential threat to global public health. This underscores the urgent need for monitoring multidrug-resistant pathogens to further curb their emergence and spread.

## 1. Introduction

Neonatal calf diarrhea (NCD) is a gastrointestinal disease affecting calves prior to weaning. It can cause hypovolemia and acidosis in calves, potentially triggering anorexia and ataxia, posing a fatal threat to young calves [1]. In addition, calves that are treated may experience a short-term or long-term decline in productivity [2], resulting in significant economic losses for the cattle industry.

Diarrhea in neonatal calves may be caused by non-infectious factors such as unprofessional husbandry and management, sudden changes in diet (diet composition or feeding method) or stress in the husbandry environment, or by infectious factors such as bacteria, viruses, and parasites. For bacteria, ETEC infection is the most common infectious cause of diarrhea in neonatal calves [3]. ETEC binds to intestinal cell receptors through adhesins and eventually delivers enterotoxins (LT and/or ST), promoting the secretion of water into the intestinal lumen and causing diarrhea. ETEC can be transmitted horizontally or vertically. Infected animals are the main hosts of ETEC, and their feces are the main source of environmental contamination by this bacterium. The spread of ETEC in animals has a “multiplier effect”, with each infected animal excreting far more bacteria than it initially ingested, especially diarrheal calves, which usually excrete 1000 mL or more of diarrheal feces containing 10^10^ ETEC/mL within 12 h, while recovered calves can continue to excrete bacteria for months. Therefore, introducing carrier animals into uninfected cattle herds is considered one of the main causes of natural outbreaks [4].

Antibiotics play an extremely important role in animal husbandry. They can not only kill bacteria and relieve symptoms but can also be used as growth promoters to promote the growth of livestock. Therefore, animal husbandry accounts for more than half of all antibiotic use worldwide. Statistics indicate that antibiotic use in animal husbandry was estimated at 131,109 tons in 2013, with projections suggesting consumption will exceed 200,000 tons by 2030 [5]. Such extensive antibiotic use inevitably leads to bacterial resistance. The overuse of antibiotics in animal agriculture particularly exacerbates the emergence and spread of bacterial resistance worldwide. In addition, the use of antibiotics in edible animals may produce antibiotic residues in livestock and poultry products (milk, eggs, meat, etc.). These residues may cause various toxic effects, such as allergies, immunopathological effects, carcinogenicity (sulfadimidine, oxytetracycline, furazolidone), mutagenicity, nephropathy (gentamicin), hepatotoxicity, reproductive disorders, and even anaphylactic shock [6].

There are many mechanisms by which bacteria develop resistance. Acquired resistance occurs when bacteria acquire resistance genes from outside the cell or mutate to develop resistance; adaptive resistance occurs when stressors appear in the living environment, and bacteria develop adaptive resistance by upregulating or downregulating the expression of certain genes. After several generations of bacterial mutation, the expression of antibiotic-inactivating enzymes and non-enzymatic pathways can be transformed into intrinsic resistance [7]. In summary, bacterial antibiotic resistance can develop through six main mechanisms: (a) efflux pump system, (b) membrane permeability alteration, (c) enzymatic degradation, (d) target site protection, (e) target site modification, and (f) expression of alternative enzymes or off-target sites [8].

This study performed whole-genome sequencing (WGS) on bovine ETEC F5- and F5-F41-positive strains in Inner Mongolia to analyze their antibiotic resistance mechanisms, providing a theoretical basis for further understanding of bovine ETEC and disease prevention and control.

## 2. Materials and Methods

### 2.1. Strain Source

ETEC F5 (F5, STa)- and F5-F41 (F5, F41, STa)-positive strains were isolated and identified by our laboratory [9] from calf diarrhea samples. Fecal samples were collected from Holstein calves aged 0–2 months at large-scale cattle farms in Hohhot, Inner Mongolia, from 2023 to 2024, with a total of 500 samples obtained. Feces were predominantly soft, with a few formed stools. Body condition was ideal, i.e., ribs were palpable but not visible, temperature, respiration and heart rate were normal, and no antibiotics were administered prior to sampling. Sampling was performed using sterile cotton swabs soaked in Nutrient Broth (Dextrose Free). Rectal swab samples were collected by gentle insertion of the swab into the anus for approximately 5–10 cm, gently rotating and touching the rectal mucosa for 3–10 s. Samples were stored at 2–8 °C for transport and sent to the laboratory for processing within 24 h. Samples were streaked onto Eosin Methylene Blue (EMB) agar plates under aseptic conditions and incubated at 37 °C for 16 h. Individual colonies exhibiting *Escherichia coli* morphology were picked and inoculated onto Nutrient Broth (NB) medium, incubated at 37 °C for 16 h, and subjected to nucleic acid extraction followed by PCR identification. The culture medium used in the study was purchased from China Beijing Land Bridge Technology Co., Ltd. (Beijing, China). (Nutrient Broth (Dextrose Free) Cat. No. CM142, EMB Cat. No. CM105, NB Cat. No. CM106). DNA extraction kits were purchased from Tiangen biochemical technology (Beijing) Co., Ltd. (Beijing, China). (Cat. No. DP302). Ultimately, ETEC F5 (F5, STa)- and F5-F41 (F5, F41, STa)-positive strains were isolated from 63 samples exhibiting characteristics consistent with *Escherichia coli*. Both ETEC strains are deposited at the China General Microbiological Culture Collection Center (CGMCC).

### 2.2. Drug Sensitivity Test

To determine the antimicrobial resistance susceptibility of ETEC F5- and F5-F41-positive strains, following the guidelines of the Clinical and Laboratory Standards Institute (CLSI, 2024) and the National Antimicrobial Resistance Surveillance System (NARMS), the Kirby–Bauer test recommended by the WHO was used to test for 21 antimicrobial agents (Supplementary Table S1), including ampicillin (AMP), ceftriaxone (CRO), ceftazidime (CAZ), cefoxitin (FOX), cefepime (FEP), meropenem (MEM), aztreonam (ATM), gentamicin (GEN), tobramycin (TOB), amikacin (AKM), streptomycin (S), kanamycin (K), ciprofloxacin (CIP), enrofloxacin (ENR), tetracycline (TET), doxycycline (DOX), chloramphenicol (CHL), trimethoprim/sulfamethoxazole (SXT), florfenicol (FFC), trimethoprim-methyl (TMP), and sulfamethoxazole (SMX). The specific procedures are as follows: Adjust the concentration of the *Escherichia coli* bacterial suspension to 1.5 × 10^8^ CFU/mL using a turbidimeter. Spread 200 µL of the suspension evenly onto a Mueller–Hinton agar plate. After incubation for 2–3 min, use sterile forceps to pick up drug susceptibility test strips and evenly place them onto the Mueller–Hinton agar plate at regular intervals. Incubate at 37 °C upside down for 16 h. The inhibition zone diameter was determined using a caliper. Determine the drug resistance of the isolates based on the interpretation criteria for the inhibition zone. Test each strain in triplicate, using *Escherichia coli* ATCC 25922 as the quality control strain. The drug susceptibility test criteria are shown in Supplementary Table S1. A strain that is resistant to one drug in three or more different antimicrobial classes is defined as a multidrug-resistant (MDR) strain [10].

### 2.3. Whole-Genome Sequencing and Bioinformatics Analysis

Nucleic acid extraction and WGS of the two isolated *Escherichia coli* strains were performed. WGS was performed by Shanghai Meiji Biotechnology Co., Ltd. (Shanghai, China).

WGS was performed using PacBio Sequel LE and Illumina sequencers (NovaSeq 6000) (Illumina, San Diego, CA, USA). For Illumina library construction, genomic DNA was fragmented into ~400 bp fragments. Fragment size distribution was identified via agarose gel electrophoresis, and library preparation was conducted using the NEXTFLEX Rapid DNA-Seg Kit. Attach A&B adapters, screen and remove adapter-autoflick fragments, and perform library enrichment. For PacBio library construction, genomic DNA was fragmented into ~10 kb fragments. End smoothing was performed according to the PacBio protocol (Pacific Biosciences, Menlo Park, CA, USA). Circular single strands were attached to both ends: each single-stranded end is connected to the positive and negative strands of the double-stranded DNA, yielding a dumbbell-like structure (“lasso”) termed SMRTBell (SMRTbellprep kit 3.0, Cold Spring Biotech Corp., Taipei, Taiwan, China).

For Illumina sequencing, the prepared library undergoes paired-end sequencing (2 × 150 bp) on an Illumina sequencer (NovaSeq 6000) (Illumina, San Diego, CA, USA). Add modified DNA polymerase and dNTPs labeled with four fluorescent dyes, incorporating only a single base per cycle; scan the reaction plate surface with a laser to read the nucleotide types incorporated during the first round of template synthesis; chemically cleave the “fluorescent group” and “terminator group” to restore 3′ stickiness and then continue synthesizing the second nucleotide; compile the fluorescence signal data collected per round to determine the sequence of the template DNA fragment. For PacBio sequencing, library single strands are annealed into loops and bound to the polymerase anchored at the bottom of the fixed ZMW (zero-mode waveguides) in the PacBio sequencer. Sequencing reaction reagents are then added. Each base pair synthesized emits a corresponding light signal that is detected. Each synthesized base is displayed as a pulse peak, and a high-resolution optical detection system enables real-time monitoring.

Raw sequencing data from Illumina instruments are stored in fastq format. To enhance the accuracy of subsequent assembly, the raw data undergo quality trimming using fastp v0.23.0. This process removes reads with low sequencing quality, high N content, or insufficient length after trimming, yielding high-quality clean data. PacBioSequele raw data are in HiFireads format. Unicycler v0.4.8 [11] is used to assemble the quality-controlled Illumina data and HiFireads. During assembly, Pilon v1.22 is employed for sequence correction. If the final assembled sequences exhibit overlapping segments of sufficient length at either end, the sequences are looped and one end of the overlap is trimmed. This process yields complete chromosome and plasmid sequences.

Genome coding sequences (CDS) were predicted using Prodigal v2.6.3 [12] for analysis. Plasmid genes were predicted using GeneMarks v4.3 [13] software. Antibiotic resistance was predicted and analyzed using the Comprehensive Antibiotic Resistance Database (CARD) (parameters: E-value ≤ 1 × 10^−5^, Identity ≥ 95%, Coverage ≥ 80%) and ResFinder 4.1 (Identity ≥ 95%, Coverage ≥ 80%) [14,15].

## 3. Results

### 3.1. Drug Sensitivity Test

**Figure 1:**
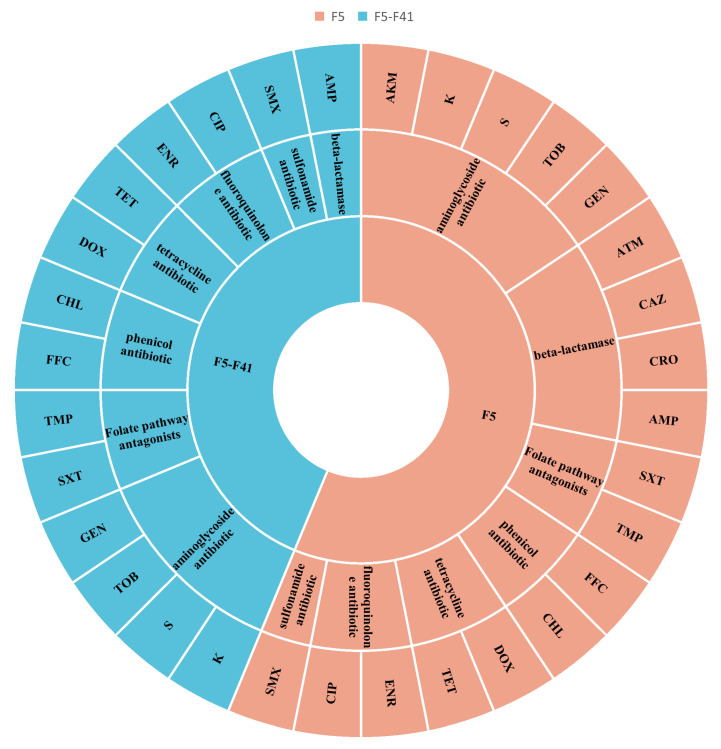
Antimicrobial resistance of ETEC F5- and F5-F41-positive strains.

The Kirby–Bauer assay results (Figure 1) showed that the ETEC F5-positive strain was resistant to 18 antibiotics, with an AMR profile of AMP–CRO–CAZ–ATM–GEN–TOB–S–K–AKM–CIP–ENR–TET–DOX–CHL–FFC–SMX–TMP–SXT. The ETEC F5-F41-positive strain was resistant to 14 antibiotics, with an AMR profile of AMP–GEN–TOB–S–K–CIP–ENR–TET–DOX–CHL–FFC–SMX–TMP–SXT.

### 3.2. Whole-Genome Sequencing of ETEC F5- and F5-F41-Positive Strains

The WGS results showed that the genomes of both strains were circular, consistent with the characteristics of bacterial genomes (Supplementary Figures S1 and S2), and the quality control data for ETEC F5- and F5-F41-positive strains were good (Supplementary Figure S3). The sequence obtained from sequencing the ETEC F5-positive strain accounted for 91.10% of the whole genome, and the sequence obtained from sequencing the ETEC F5-F41-positive strain accounted for 91.36% of the whole genome. The ETEC F5-positive strain gene length was 5,110,765 bp, containing 1 chromosome and 3 plasmids, with an average GC content of 50.47%. The F5-F41-positive strain gene length was 5,240,155 bp, containing 1 chromosome and 5 plasmids, with an average GC content of 50.65%.

### 3.3. Carrying Antibiotic Resistance Genes

**Figure 2:**
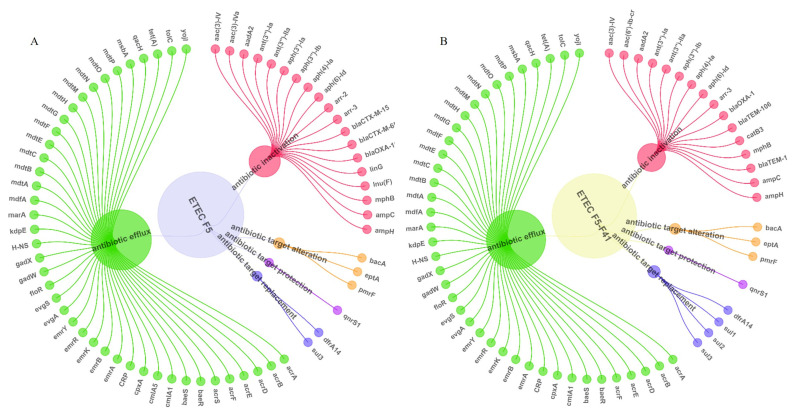
ETEC F5- and F5-F41-positive strains antimicrobial resistance genes. (**A**): antimicrobial resistance gene of ETEC F5 positive strain, (**B**): antimicrobial resistance gene of ETEC F5-F41 positive strain; Green circles represent bacterial efflux pumps, pink circles represent antibiotic inactivation, orange circles represent antibiotic target alteration, purple circles represent antibiotic target protection, and blue circles represent antibiotic target displacement.

Analysis of the combined results from the Comprehensive Antibiotic Resistance Database (CARD) and ResFinder (Figure 2) revealed 11 classes of antibiotic resistance genes in the ETEC F5- and F5-F41-positive strains, including β-lactamases (*blaTEM*, *blaCTX-M*), aminoglycosides (*AAC(3)-IV*, *aadA*), sulfonamides (*sul1*, *sul2*), fluoroquinolones (*qnrS*, *aac(6′)-Ib-cr*), chloramphenicols (*catB3*, *cmlA1*), tetracyclines (*tet(A)*), rifamycins (*arr-2*, *arr-3*), lincosamides (*linG*, *lnu(F)*), macrolides (*mphB*), peptides (*bacA*, *eptA*), and diaminopyrimidines (*dfrA14*). In addition, a variety of bacterial efflux pump-related genes were detected, including members of the RND (Resistance-Nodulation-Division) superfamily, MFS (Major Facilitator Superfamily), SMR (Small Multidrug Resistance) family, and ABC (ATP-Binding Cassette) superfamily (such as AcrAD-TolC, AcrAB-TolC, *mdtM*, EmrAB-TolC, *qacH*, *YojI*, etc.). A total of 74 drug resistance genes were identified in the two strains, belonging to five categories of drug resistance mechanisms: antibiotic inactivation (24), antibiotic efflux (42), target substitution (4), target alteration (3), and target protection (1). Chromosomal mutations related to resistance to fluoroquinolone antibiotics were also detected, namely: the replacement of serine at position 83 of the *gyrA* gene with leucine (S83L), the replacement of aspartic acid at position 87 with asparagine (D87N), and the replacement of serine at position 80 of the *parC* gene with isoleucine (S80I).

### 3.4. Relationship Between Antibiotic Resistance Genotype and Phenotype

The resistance genes *blaCTX-M-15*, *blaCTX-M-65*, *blaTEM-106*, *ampC*, *ampH*, *blaOXA-10*, *blaTEM-1*, *acc(3)-IV*, *aadA2*, *APH(6)-Id*, *aph (3′)-Ia*, *qnrS1*, *tet(A)*, *cmlA1*, *cmlA5*, *floR*, catB3, *sul1*, *sul2*, *sul3*, and *dfrA14* correspond to antibiotics, third-generation cephalosporins, aminoglycosides, fluoroquinolones, tetracyclines, chloramphenicols, sulfonamides, and folic acid pathway antagonists, respectively, and are consistent with the phenotypes (Table 1 and Table 2).

## 4. Discussion

NCD causes a large loss of water and electrolytes in calves, leading to dehydration and acid-base imbalance, which can result in death in severe cases. Even after treatment, the growth performance of calves is severely affected. In addition, 60% of diarrhea in 4-day-old calves is related to ETEC, and diarrhea caused by ETEC is more severe than diarrhea caused by rotavirus and coronavirus [4]. Antibiotics are commonly used to treat diarrhea in clinical practice, but 30–90% of antibiotics are excreted in feces or urine as the original drug or active metabolites, which leads to the contamination of the environment by drug-resistant bacteria and increases the risk of contact with humans [16]. At the same time, many antimicrobial drugs used in livestock production and treatment are closely related to human medication, which makes the use of antimicrobial drugs in animal husbandry one of the important factors leading to the emergence and spread of drug-resistant bacteria. Therefore, it is urgent to establish a monitoring program to monitor multidrug-resistant *Escherichia coli* that may be transmitted from food animals to humans [17].

This study performed WGS on ETEC F5-positive and F5-F41-positive strains from Inner Mongolia, China. In our investigations of calf diarrhea on farms, approximately 12–25% of samples tested positive for F5 and F5-F41 ETEC strains, indicating the presence of an epidemic within local cattle populations. *BlaCTX-M-15*, *blaCTX-M-65*, and *blaOXA-10* were present in the ETEC F5-positive strains, while *blaTEM-1*, *blaTEM-106*, and *blaOXA-1* were present in the F5-F41-positive strains. *blaCTX-M-15* and *blaCTX-M-65* belong to the CTX-M-1 and CTX-M-9 groups, respectively. *blaCTX-M-65* has been described in bovine *Escherichia coli* from China, the United States, the Netherlands, and Canada. *blaCTX-M-15* is one of the most common genotypes in the bla CTX-M family and has been found in all major ecological niches (humans, animals, and the environment) [18]. *blaTEM-1* is the first plasmid and transposon-mediated broad-spectrum β-lactamase, which has spread worldwide and is now present in many bacteria of the Enterobacteriaceae family [19]. However, the ultra-broad-spectrum *blaTEM-106* has only been detected in broiler farms in Belgium [20] and has rarely been reported in foodborne animals. The β-lactamase genes carried by the 2 enterotoxin-producing *Escherichia coli* strains in this study are basically consistent with the global prevalence.

In this study, a total of 10 drug resistance genes that induce resistance to aminoglycoside drugs through enzyme inactivation were detected in ETEC F5- and F5-F41-positive strains, including aminoglycoside phosphotransferase, aminoglycoside acetyltransferase and aminoglycoside nucleoside transferase. In *Escherichia coli*, APH(6)-Id, encoded by the *strB* gene, is one of the most common aminoglycoside phosphotransferases worldwide, while the nucleotide transferase ANT(3″), encoded by the *aadA* gene, is common in Gram-negative bacteria [21]. In addition, the aminoglycoside N-acetyltransferase AAC(3)-IV mainly leads to resistance to gentamicin and a low degree of kanamycin (KAN) resistance, while the aminoglycoside O-phosphotransferase encoded by *aph(3′)-Ia* can produce kanamycin resistance [22]. Finally, the *aac(6′)-Ib-cr* gene was also found in ETEC F5- and F5-F41-positive strains, which reduces susceptibility to ciprofloxacin through piperazine N-acetylation [23].

The most common resistance mechanism to tetracycline antibiotics is through efflux pumps that expel antibiotics from the cytoplasm. Nine efflux genes (*tet(A)*, *tet(B)*, *tet(C)*, *tet(D)*, *tet(E)*, *tet(G)*, *tet(J)*, *tet(L)*, and *tet(Y)*) have been identified in *Escherichia coli* [21,24]. Among them, the *tet(A)* gene has been reported in human and animal *Escherichia coli* isolates from different countries and is commonly found in animal-derived *Escherichia coli* isolates [25]. The *tet(A)* tetracycline resistance gene was detected in ETEC F5- and F5-F41-positive strains, consistent with the above reports.

The low permeability of the outer membrane and the multidrug efflux system of *Escherichia coli’* lead to its resistance to most macrolides [26,27]. Therefore, macrolides are generally not considered for the treatment of bacterial infections caused by *Escherichia coli*. However, given the ongoing spread of widely resistant *Escherichia coli*, macrolides may be a viable alternative for treating diarrhea caused by *Escherichia coli* [28]. CARD and ResFinder identified the *mphB* gene in 2 ETEC strains, which encodes macrolide 2′-phosphotransferase II, leading to resistance to 14-membered and 16-membered macrolide antibiotics through phosphorylation [26,29]. Previous studies have identified different macrolide resistance genes in *Escherichia coli* [30,31], suggesting that *Escherichia coli* may serve as a reservoir of macrolide resistance genes [32] and further spread macrolide resistance genes globally.

Fluoroquinolones play an important role in combating bacterial infections in humans and animals. In this study, *gyrA* and *parC* mutations were detected in the chromosomes of ETEC F5- and F5-F41-positive strains. Studies have shown that a single mutation in the *gyrA* gene may lead to resistance to quinolones, but further mutations in the *gyrA* and/or *parC* genes are required to develop resistance to fluoroquinolones [21,33]. Secondly, the most common mutations in GyrA in *Escherichia coli* occur at codons 83 and 87, while most ParC mutations occur at codons 80 and 84 [34], which is consistent with the findings in this study. The plasmid-mediated quinolone resistance protein QnrS1 was also found. This resistance protein does not lead to high levels of resistance to fluoroquinolones, but rather reduces sensitivity to the antimicrobial agents. However, it can increase resistance to these antimicrobials by coexisting with chromosomally encoded resistance mechanisms [21,33].

The chloramphenicol resistance genes *catB3*, *cmlA1*, *cmlA5*, and *floR* were found in the plasmids of ETEC F5- and F5-F41-positive strains. Since 2002, China has banned the use of chloramphenicol in animal production by any means. The chloramphenicol resistance genes found in this study may be related to the use of florfenicol in animal husbandry [35]. These resistance genes, located on mobile elements, are important conditions for their rapid and effective spread among the same or different bacterial species.

Sulfonamides are usually used alone or in combination with trimethoprim to show significant bactericidal effects on susceptible bacteria. The mechanism that determines sulfonamide resistance mainly includes the dihydrofolate synthase sul gene, which alters the drug binding site. Currently, *sul 1*, *sul 2*, *sul 3*, and *sul 4* have been detected in *Escherichia coli*. This study detected *sul 1*, *sul 2*, and *sul 3*. According to reports, the most common sulfonamide resistance genes in *Escherichia coli* are *sul 1* and *sul 2* [33], while *sul 3* gene diversity is low. Some *sul 3* genes are located on chromosomes [36], which affects their spread. However, the detection frequencies of *sul 1*, *sul 2*, and *sul 3* genes reported in different studies are also different. This regional difference in gene distribution may be due to the significant differences in the intensity of sulfonamide use in different regions and the heterogeneity of antimicrobial drug regulatory policies of different governments. For trimethoprim, the mechanism that determines its resistance is mainly the dihydrofolate reductase encoded by the *dfr* gene, which alters the drug binding site. Based on the length of the N-terminus of the enzyme and the level of resistance they confer, they can be divided into two families: family A is encoded by the *dfrA* gene, and family B is encoded by the *dfrB* gene [37]. In this study, the *dfrA14* gene carried by the plasmid was detected in both ETEC F5- and F5-F41-positive strains. It is known that this gene exists in the form of a gene cassette in the integrin or outside the integrin, but it is inserted into the *strA* gene carried by the plasmid [21].

The *mcr* gene is a plasmid-mediated resistance gene that encodes an enzyme belonging to the phosphoethanolamine transferase family that synthesizes pEtN and couples it to lipid A [38], thereby leading to the horizontal spread of colistin resistance. The *mcr* gene was not found in this study, which may be due to the Chinese government’s ban on the use of colistin as an animal growth promoter [39].

Bacterial genomes typically encode multiple efflux pumps. These transmembrane protein complexes release antibiotics from the cell via active efflux mechanisms, reducing the intracellular drug accumulation concentration and thus achieving antibiotic resistance. This plays an important role in the adaptive survival of bacteria under high concentrations of antibiotic selection pressure. In this study, we found multiple efflux pump genes in ETEC F5- and F5-F41-positive strains. These efflux pumps pump out multiple antibiotics due to overexpression caused by mutations in local repressor genes or activation by global transcriptional regulators (such as MarA, BaeSR, and EvgAS in *Escherichia coli*) [40]. In addition, there are many types of efflux pumps, and their functions have substrate cross-reactivity: on the one hand, a single antibiotic can be recognized and effluxed by a variety of structurally different efflux pumps (for example, fluoroquinolone antibiotics can be pumped out by RND bacterial efflux pumps (such as AcrAB-TolC) and transported by MFS bacterial efflux pumps (such as QepA)); on the other hand, a single efflux pump often has broad-spectrum substrate specificity and can recognize and efflux antibiotics that are highly heterogeneous in structure and chemical properties (such as AcrB can simultaneously efflux hydrophilic β-lactamase antibiotics and hydrophobic macrolide antibiotics) [41]. This also leads to a very important problem: cross-resistance, as contact with any substance that is a substrate characteristic of the pump will lead to overexpression of the pump, thereby generating cross-resistance to all other substrates of the pump [40]. This “cross-reactivity” is not limited to antibiotics used in clinical applications, but also includes disinfectants and antiseptics (such as MdtM, which can efflux disinfectants, antiseptics, lincomycin and fluoroquinolone antibiotics). This characteristic enables drug-resistant bacteria to develop adaptive tolerance to a variety of detergents and disinfectants (including commonly used bactericides such as triclosan), thereby enhancing their ability to survive and adapt in complex environments where high concentrations of antibiotics and disinfectants coexist. This poses a serious threat to the sustainable, healthy, and green development of animal husbandry and to human public health and safety.

The results of the drug susceptibility test showed that the ETEC F5- and F5-F41-positive strains were multidrug-resistant bacteria. The third-generation cephalosporins (ceftriaxone (CRO) and ceftazidime (CAZ)) and fluoroquinolone antibiotics (ciprofloxacin (CIP)) included in the study are listed as human highest priority critically important antimicrobial (HP-CIAs) by the World Health Organization (WHO). These drugs are of great significance for the treatment of multidrug-resistant bacterial infections in clinical practice.

A study in Egypt showed that its ETEC strains isolated from diarrheal calves were resistant to neomycin, gentamicin, streptomycin, and amikacin [42]. A study in Iran showed that its ETEC strains isolated from calf diarrhea were resistant to streptomycin, sulfonamides, gentamicin, ampicillin, and trimethoprim [43]. A study in the United States showed that its bovine ETEC isolates carried resistance genes for β-lactamases, tetracyclines, sulfonamides, and aminoglycosides [44]. There are many reasons for the differences in antimicrobial resistance. First, countries and regions with sufficient scientific and technological experience can quickly detect and identify the antimicrobial resistance of pathogens, thereby guiding precise drug use and reducing the use of broad-spectrum antibiotics. On the contrary, some underdeveloped countries and regions usually rely on empirical drug use and tend to choose broad-spectrum antibiotics. Second, there is heterogeneity in government regulation, and different regulatory schemes lead to different antimicrobial resistance. Finally, different countries and regions have different understandings of antibiotics. Countries and regions with poor knowledge of antibiotics often have antibiotic abuse, and long-term antibiotic abuse leads to an increase in the abundance of antibiotic resistance genes in natural microbial communities and spreads among microorganisms at the gene level, enabling them to develop antibiotic resistance outside their original metabolic networks [45].

Antibiotics in animal feces can pollute soil and water sources, and antimicrobial drugs released into aquatic and soil ecosystems can promote the growth of antifungal microorganisms, leading to the emergence of antibiotic resistance genes in the ecosystem [46]. At the same time, aerosols generated in areas with high antibiotic resistance can indirectly spread antibiotic-resistant bacteria to humans, animals and the entire environment. Antibiotic-resistant pathogens can indirectly cause humans to acquire antibiotic resistance through the consumption of animals or highly antibiotic-resistant environments (such as hospitals, feces, wastewater, etc.) [47]. Therefore, the increase in foodborne infections related to antibiotic-resistant pathogens and the spread of antibiotic resistance are major concerns for developed and developing countries worldwide [48].

The genomic data obtained from the F5 and F5-F41 strains in this study provide traceable and comparable genomic references for research on bovine ETEC resistance. This facilitates comparative analysis of resistance gene lineages, inference of transmission pathways for resistance determinant clusters, and monitoring of plasmid-mediated resistance at regional and transregional scales. Notably, the detection of multiple genes associated with resistance to third-generation cephalosporins and fluoroquinolones provides molecular epidemiological evidence for assessing zoonotic antimicrobial resistance risks. This finding suggests that empirical use of relevant antibiotics should be strictly restricted in calf diarrhea management, promoting precision medication strategies based on antimicrobial susceptibility testing and molecular detection results to reduce selection pressure and dissemination risks for resistant strains. This study systematically elucidates the molecular basis for the coexistence of multiple resistance mechanisms in ETEC strains, including antibiotic inactivation, efflux pump-mediated resistance, target alteration, and protection. These findings provide research directions and a data foundation for future investigations into the mechanisms underlying ETEC resistance development, the evaluation of alternative or adjunctive therapeutic strategies involving efflux pump inhibitors, and the exploration of relationships between resistance genes, virulence factors, and adaptive evolution. It holds practical significance for refining prevention and control strategies against neonatal NCD and promoting the rational use of antimicrobial agents in animal husbandry.

Currently, there are few research reports on bovine ETEC and its antibiotic resistance. With the rising incidence of diarrhea in neonatal calves and the increasing status of edible animals as reservoirs of pathogens and antibiotic resistance genes, we urgently need to monitor multidrug-resistant pathogens to safeguard the high-quality development of cattle farming and its livestock products, and further protect public health and food safety.

## 5. Conclusions

This study performed WGS on ETEC F5- and F5-F41-positive strains from Inner Mongolia and analyzed their drug resistance and resistance genes. The results showed that both strains were multidrug-resistant, suggesting that establishing an *Escherichia coli* monitoring system and reducing the use of veterinary antibiotics will help control *Escherichia coli* infection and the emergence of multidrug-resistant *Escherichia coli*.

## Figures and Tables

**Table 1:** Antimicrobial resistance genotypes, phenotypes, and bacterial efflux pump characteristics of ETEC F5 strain.

Isolate	Antibiotic Types	AMR Genes	AMR Phenotype
ETEC F5	β-lactamase	*blaCTX-M-15* *blaCTX-M-65* *blaOXA-10* *ampC* *ampH*	AMP CRO CAZ ATM
	aminoglycoside antibiotic	*acc(3)-IV* *aac(3)-IVa* *aadA2* *ant(3″)-Ia* *ant(3″)-IIa* *aph(3′)-Ia* *aph(3″)-Ib* *aph(4)-Ia* *aph(6)-Id*	GEN TOB S K AKM
	fluoroquinolone antibiotic	*qnrS1*	CIP ENR
	tetracycline antibiotic	*tet(A)*	TET DOX
	phenicol antibiotic	*cmlA1* *cmlA5* *floR*	CHL FFC
	sulfonamide antibiotic	*sul3*	SMX
	folate pathway antagonists	*dfrA14*	TMP SXT
	macrolide antibiotic	*mphB*	N/A *
	peptide antibiotic	*bacA* *eptA* *pmrF*	N/A
	lincosamide antibiotic	*linG* *lnu(F)*	N/A
	rifamycin antibiotic	*arr-2* *arr-3*	N/A
	bacterial efflux pumps	*acrA*, *acrB*, *acrD*, *acrE*, *acrF*, *acrS*, *baeR*, *baeS*, *cpxA*, *CRP*, *emrA*, *emrB* *emrK*, *emrR*, *emrY*, *evgA*, *evgS*, *gadW*, *gadX*, *H-NS*, *kdpE*, *marA* *mdfA*, *mdtA*, *mdtB*, *mdtC*, *mdtE* *mdtF*, *mdtG*, *mdtH*, *mdtM*, *mdtN* *mdtO*, *mdtP*, *msbA*, *qacH*, *tolC* *yojI*	N/A

* N/A: not applicable.

**Table 2:** Antimicrobial resistance genotypes, phenotypes, and bacterial efflux pump characteristics of ETEC F5-F41 strain.

Isolate	Antibiotic Types	AMR Genes	AMR Phenotype
ETEC F5-F41	β-lactamase	*blaOXA-1* *blaTEM-106* *blaTEM-1* *ampC* *ampH*	AMP
	aminoglycoside antibiotic	*aac(3)-IV* *aac(6′)-Ib-cr* *aadA2* *ant(3″)-Ia* *ant(3″)-IIa* *aph(3″)-Ib* *aph(4)-Ia* *aph(6)-Id*	GEN TOB S K
	fluoroquinolone antibiotic	*qnrS1*	CIP ENR
	tetracycline antibiotic	*tet(A)*	TET DOX
	phenicol antibiotic	*cmlA1* *catB3* *floR*	CHL FFC
	sulfonamide antibiotic	*sul1* *sul2* *sul3*	SMX
	folate pathway antagonists	*dfrA14*	TMP SXT
	macrolide antibiotic	*mphB*	N/A *
	peptide antibiotic	*bacA* *eptA* *pmrF*	N/A
	rifamycin antibiotic	*arr-3*	N/A
	bacterial efflux pumps	*acrB*, *acrD*, *acrE*, *acrF*, *baeR*, *baeS* *cpxA*, *CRP*, *emrA*, *emrB*, *emrK*, *emrR* *emrY*, *evgA*, *evgS*, *gadW*, *gadX* *H-NS*, *kdpE*, *marA*, *mdtA*, *mdtB* *mdtC*, *mdtE*, *mdtF*, *mdtG*, *mdtH* *mdtM*, *mdtN*, *mdtO*, *mdtP*, *msbA* *tolC*, *yojI*, *acrA*, *mdfA*, *qacH*	N/A

* N/A: not applicable.

## Data Availability

The original contributions presented in this study are included in the article/Supplementary Materials. Further inquiries can be directed to the corresponding authors.

## Supplementary Materials

The following supporting information can be downloaded at: https://www.mdpi.com/article/10.3390/ani16010151/s1, Figure S1: ETEC F5 positive strains Genome Circos Map; Figure S2: ETEC F5-F41 positive strains Genome Circos Map; Figure S3: Statistical analysis results of quality control data for ETEC F5 and F5-F41 positive strains; Table S1:Drug susceptibility testing standards.
