# Supplementary material for: Whole-Genome Sequencing and Antimicrobial Resistance Analysis of Enterotoxigenic Escherichia coli F5 and F5-F41 Strains Isolated from Neonatal Calves in Inner Mongolia, China
Source: Animals (Basel). 2026 Jan 5;16(1):151. doi: 10.3390/ani16010151 (PMC12785056; doi:10.3390/ani16010151)
Supplement: Supplementary file 1 [file animals-16-00151-s001.zip › animals-4012397-supplementary.pdf]

Whole-genome sequencing and antimicrobial resistance analysis of enterotoxigenic *Escherichia coli* F5 and F5-F41 positive strains in Inner Mongolia, China

Supplementary Material

1 Supplementary Figures and Tables

1.1 Supplementary Figures

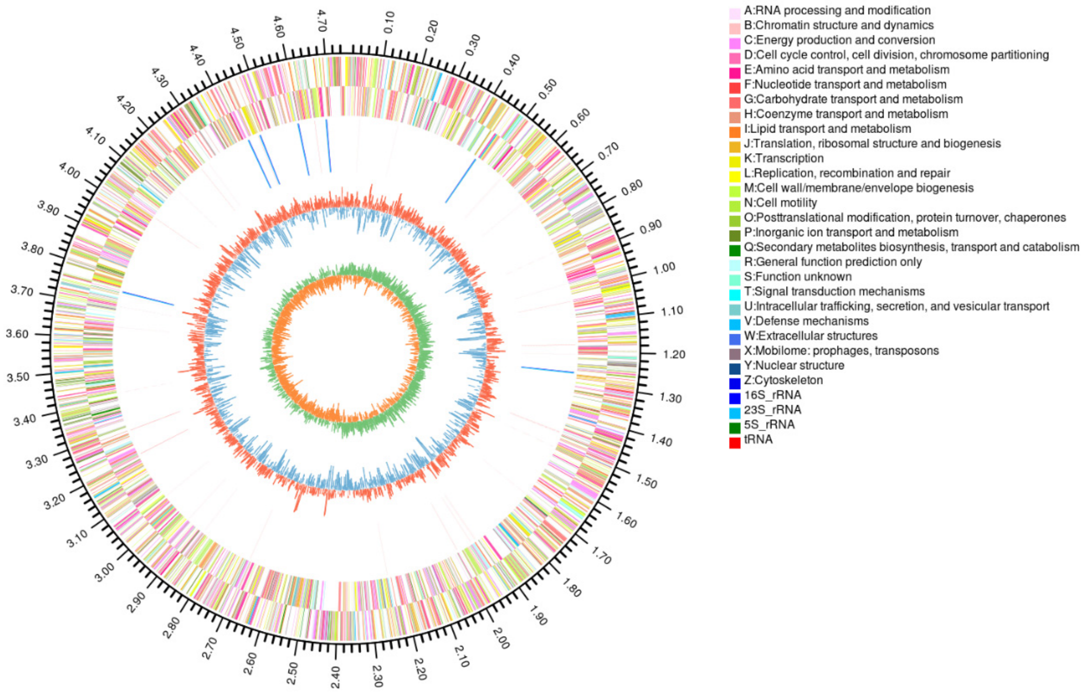

Supplementary Figure S1. ETEC F5 positive strains Genome Circos Map

Note: The outermost circle of the circle diagram is the identification of genome size; the second and third circles are CDS on positive and negative strands, and different colors indicate the functional classification of different COGs of CDS; the fourth circle is rRNA and tRNA; the fifth circle is GC content; the innermost circle is the GC-Skew value.

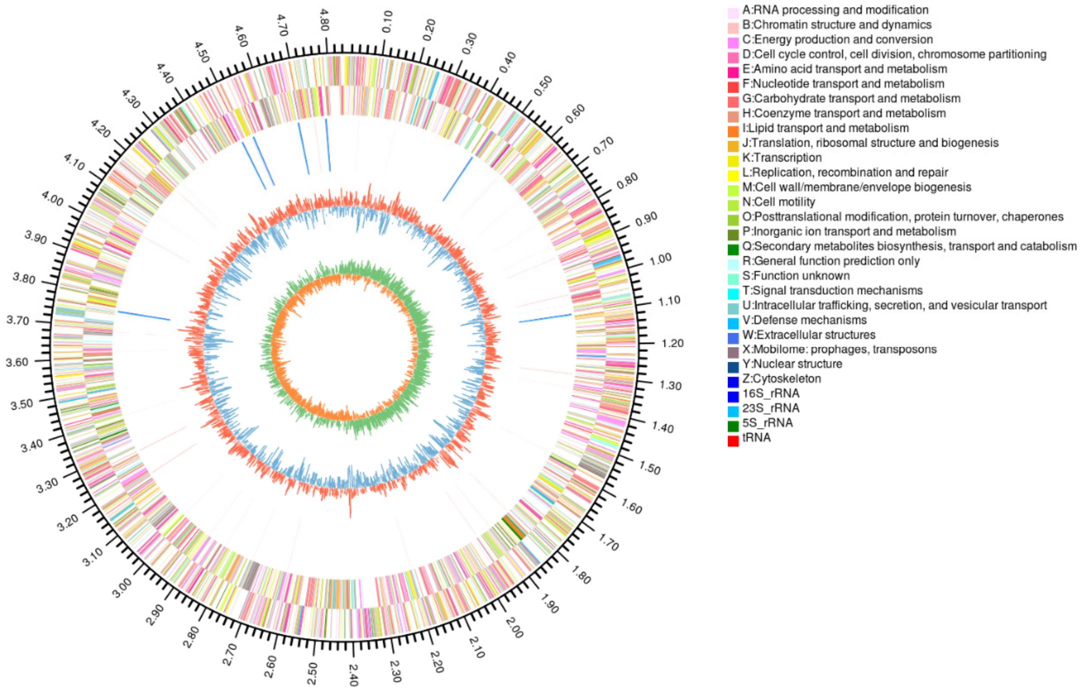

Supplementary Figure S2. ETEC F5-F41 positive strains Genome Circos Map

Note: The outermost circle of the circle diagram is the identification of genome size; the second and third circles are CDS on positive and negative strands, and different colors indicate the functional classification of different COGs of CDS; the fourth circle is rRNA and tRNA; the fifth circle is GC content; the innermost circle is the GC-Skew value.

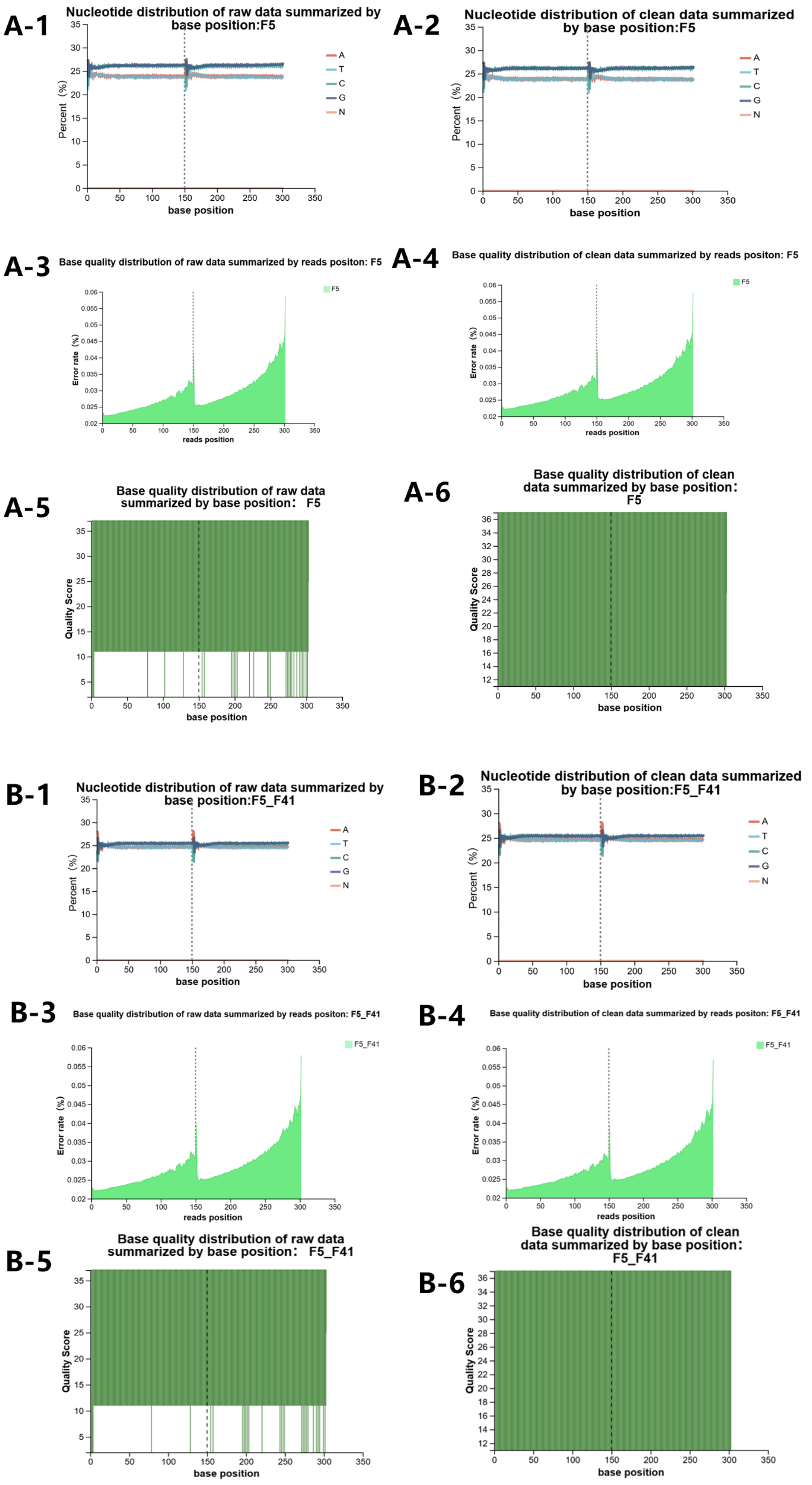

Supplementary Figure S3. Statistical analysis results of quality control data for ETEC

F5 and F5-F41 positive strains

Note: A1-2、B1-2: Base composition distribution diagrams of ETEC F5 and F5-F41 positive strains before and after quality control. The horizontal axis is the base coordinate of reads, representing the sequential arrangement of bases from the 5' to the 3' end of the reads; the vertical axis is the percentage of A, C, G, T, and N bases in all reads at this sequencing position, with different bases represented by different colors.

A3-4、B3-4: Pre-quality control base error rate distribution of ETEC F5 and F5-F41 positive strains. The horizontal axis represents the base position of the reads, indicating the arrangement of bases from the 5' to 3' ends; the vertical axis represents the average error rate (%) of all reads at that position. The first half shows the error rate distribution of the first end of the paired-end sequencing reads, and the second half shows the error rate distribution of the other end. The horizontal axis represents the base position of the reads, and the vertical axis represents the single-base error rate.

A5-6、B5-6: Box plot of base quality before quality control for ETEC F5 and F5-F41 positive strains. The horizontal axis represents the base position of reads, indicating the arrangement of bases from the 5' to 3' ends of the reads; the vertical axis represents the Q value of all reads at that site. The first half shows the Q value distribution of the first end of the paired-end sequencing reads, and the second half shows the Q value distribution of the other end of the sequencing reads.

1.2 Supplementary Tables

Supplement Table S1. Drug susceptibility testing standards

**Antibiotic** types	Antibiotics	Drug concentration	S	I	R
**beta-lactamase**	AMP	10μg	≤13	14-16	≥17
	CRO	30μg	≤19	20-22	≥23
	CAZ	30μg	≤17	18-20	≥21
	FOX	30μg	≤14	15-17	≥18
	FEP	30μg	≤18	19-24	≥25
	MEM	10μg	≤19	20-22	≥23
	ATM	30μg	≤15	16-21	≥22
**aminoglycoside antibiotic**	GEN	30μg	≤17	18-20	≥21
	TOB	10μg	≤12	13-16	≥17
	S	10μg	≤11	12-14	≥15
	K	30μg	≤13	14-17	≥18
	AKM	30μg	≤14	15-16	≥17
**fluoroquinolone antibiotic**	CIP	5μg	≤21	22-25	≥26
	ENR	5μg	≤16	17-20	≥21
**tetracycline antibiotic**	TET	30μg	≤11	12-14	≥15
	DOX	30μg	≤10	11-13	≥14
**phenicol antibiotic**	CHL	30μg	≤12	13-17	≥18

	FFC	30μg	≤14	15-18	≥19
**sulfonamide antibiotic**	SMX	300μg	≤12	13-16	≥17
**folate pathway**	TMP	5μg	≤10	11-15	≥16
**antagonists**	SXT	1.25/23.75 μg	≤10	11-15	≥16
